# Increased soluble IL-7 receptor concentrations associate with improved IL-7 therapy outcomes in SIV-infected ART-treated Rhesus macaques

**DOI:** 10.1371/journal.pone.0188427

**Published:** 2017-12-19

**Authors:** Amanda K. Steele, Lorna Carrasco-Medina, Donald L. Sodora, Angela M. Crawley

**Affiliations:** 1 Center for Infectious Disease Research, Seattle, WA, United States of America; 2 Collegiate Peaks Science Writing, Denver, CO, United States of America; 3 The Ottawa Hospital–General Campus, Division of Infectious Diseases, Ottawa, ON, Canada; 4 The Ottawa Hospital Research Institute, Chronic Disease Program, Ottawa, ON, Canada; 5 University of Ottawa, Dept. Biochem., Microbiol., and Immunol., Ottawa, ON, Canada; 6 Carleton University, Dept. Biol., Ottawa, ON, Canada; University of Pittsburgh Centre for Vaccine Research, UNITED STATES

## Abstract

The use of interleukin-7 (IL-7) as an immunorestorative therapeutic has proven effective in HIV infection, cancer and bone marrow transplantation. Mediating its activity through membrane-bound IL-7 receptor α (mCD127), IL-7 therapy increases T-cell numbers and survival. A soluble form, sCD127, is found in plasma, and we have previously identified increased plasma sCD127 concentrations in HIV infection. Furthermore, patients with high sCD127 exhibited the best viral control, implicating a role for IL-7 or sCD127 directly in improved virologic/immunologic outcomes. The role of the cytokine IL-7 in elevating sCD127 levels was addressed here through assessment of retrospective samples obtained from SIV-infected antiretroviral (ART)-treated Rhesus macaques. IL-7 was administered in clustered weekly doses, allowing for an assessment prior, during and following IL-7 administration. The levels of sCD127 remained relatively unchanged during both early SIV infection and following initiation of ART. However, treatment with IL-7 increased sCD127 concentrations in most animals, transiently or persistently, paralleling increased T-cell numbers, correlating significantly with CD8^+^ T-cell levels. In addition, proliferating CD4^+^ or CD8^+^ T-cells (measured by Ki67) increased in association with elevated sCD127 concentrations. Finally, a high concentration of sCD127 in IL7-treated animals was associated with increased retention of T-cells (measured by BrDU). In addition, a lack, or loss of viral control was associated with more pronounced and frequent elevations in plasma sCD127 concentrations with IL-7 therapy. In summary, plasma sCD127 levels in SIV-infected ART-treated macaques was associated with therapeutic IL-7 administration, with higher sCD127 levels in macaques demonstrating the best T-cell responses. This study furthers our knowledge regarding the interrelationship between increased IL-7 levels and elevated sCD127 levels that may have implications for future IL-7 immunotherapeutic approaches in HIV-infected patients.

## Introduction

Interleukin-7 (IL-7) regulates the homeostasis of mature T-cells by inducing cell proliferation and promoting cell survival by altering the balance between pro- and anti- apoptotic proteins. IL-7 has been shown to increase the expression of anti-apoptotic proteins (e.g. Bcl-2) and decrease pro-apoptotic protein expression (e.g. Bad and Bax)[1–5]. Given its role in T-cell homeostasis, IL-7 immunotherapy has been widely applied to diseases in which T-cells may play a role. Therapeutic administration of IL-7 has been evaluated for the treatment of cancer[6], chronic HIV infection[7, 8] and transplantation[9] in which T-cell reconstitution is required. IL-7 therapy is also under consideration for the treatment of bacterial-mediated sepsis and parasitic infections[10–13]. Clinical trials conducted in bone-marrow transplant and cancer patients, indicated that IL-7 therapy increased T-cell expansion, survival, thymic output, and T-cell receptor repertoire diversity[14, 15]. In an attempt to address T-cell deficiencies in human immunodeficiency virus (HIV) infection, studies evaluated the effects of IL-7 therapy in humans and the non-human primate model of simian immunodeficiency virus (SIV). Findings suggest that IL-7 immunotherapy has a net positive effect with regard to increasing T-cell numbers and survival[7, 8] and in SIV-infected Rhesus macaques, IL-7 therapy aided to overcome IFN-α treatment-induced lymphopenia[16].

Regulation of IL-7 activity on T-cells can occur by the altered expression of its receptor, composed of the IL-7 receptor α (CD127) and the IL-2 receptor α chains[17], the latter of which exists in a membrane-bound form (mCD127) and as a soluble receptor (sCD127). The expression of mCD127 varies in thymic development and T-cell responses, with pronounced downregulation on effector cells. We also know that mCD127 expression on T-cells is reduced by IL-7, paralleling cell cycle progression, and that CD8^+^ T-cells subsequently release sCD127[18]. Expression of mCD127 on T-cells is significantly reduced in HIV infection and recovers to near normal concentrations with highly active antiretroviral therapy (HAART)[19–21]. In addition, we and others have found that plasma sCD127 concentrations are increased in HIV infection, are associated with a more rapid HIV disease progression and plasma sCD127 concentrations are not lowered with successful HAART treatment[18, 22–25]. Whether the release of this receptor from cells accounts in part for the reduction in mCD127 in health and disease, and hence a means of regulating IL-7 signaling to a cell, remains to be determined. It was thought that sCD127 may act as a decoy receptor and suppressor of cytokine signaling, however we and others have recently demonstrated that pre-incubating T-cells with recombinant sCD127 enhanced IL-7 activity in murine models and cultures of human or mouse T-cells[26, 27]. Carini et al., detected more sCD127 in the culture supernatants of PBMC collected from HIV^+^ individuals with undetectable HIV-specific CTL activity compared to those with detectable CTL activity[24], suggesting that sCD127 may influence CTL function *in vivo*. The factors and mechanisms mediating the release of sCD127 are not entirely understood, but it is known that IL-7 induces the release of sCD127 from CD8^+^ T-cells[8] while mCD127 is downregulated[18], and this may be mediated by proteolytic cleavage of the receptor (Angel, J.B., personal communication, unpublished)[28]. The role of sCD127 in the effects of IL-7 immunotherapy is not known.

Based on our previous research which identified an increase in plasma sCD127 in HIV infection, and our preliminary understanding of how IL-7 induces sCD127 release from T-cells[18, 29], we questioned the effect of IL-7 therapy on plasma sCD127 concentrations, and whether the soluble receptor was associated with the therapeutic effects of IL-7 on T-cells in HIV infection. The Rhesus macaque/SIV infection model is one of the mainstays of HIV research. Disease progression in macaques is closely analogous to disease progression in humans and is characterized by high viral loads, rapid mucosal T-cell loss, gradual peripheral T-cell depletion, and eventual progression to simian AIDS[30, 31]. In this model, studies have revealed how the progressive loss of certain T-cells subsets, or their impaired function, contributes to AIDS progression. Previously, IL-7 has been tested as an immunotherapeutic in SIV-infected macaques being treated with anti-retroviral drugs, analogous to chronically infected HIV patients, and resulted in the expansion of relevant CD4^+^ and CD8^+^ T-cell populations[32, 33]. Leone A. (now Steele, A.) et al. demonstrated that a clustered dosing regimen of glycosylated recombinant simian IL-7 (rsIL-7gly) induced the proliferation of CD4^+^ and CD8^+^ central memory and naïve cells, increased T-cell survival and sustained elevation of peripheral CD4^+^ T-cell counts in ART-treated SIV-infected Rhesus macaques[34]. Clinical studies in HAART-treated HIV^+^ patients have had similar outcomes[7, 8]. Therefore, we retrospectively quantified sCD127 concentrations in plasma samples collected from our previously published study[34] to determine for the first time whether sCD127 release is altered in early SIV infection, (2) whether antiretroviral (ART) reversed any such change, (3) to test the effect of exogenous IL-7 on the release of its receptor *in vivo*, and (4) to test whether plasma sCD127 concentrations were associated with the therapeutic effects of IL-7 on T-cells in the treatment of chronic viral infection. This study provides key insights regarding increased IL-7 levels and elevated sCD127 levels that may have implications for future IL-7 immunotherapeutic approaches in HIV-infected patients.

## Methods

### Animal research ethics statement

The animals in this study were housed at the Oregon National Primate Research Center (ONPRC). This work received prior approval by the Institutional Animal Care and Use Committee (IACUC) of ONPRC IACUC approved protocol (IACUC #492–02: “Immune Reconstitution in SIV Infected Macaques”). IACUC proposals include a written scientific justification for any exclusions from some or all parts of the plan. In addition, this study was performed in strict accordance with **1)** the recommendations in the Guide for the Care and Use of Laboratory Animals of the National Institutes of Health, a national set of guidelines in the U.S., **2)** international recommendations detailed in the Weatherall Report (2006) and **3)** in accordance with the ONPRC Institutional Animal Care and Usage Committees, accredited by American Association of Accreditation of Laboratory Animal Care. Rhesus macaques are fed standard monkey chow (Jumbo Monkey Diet 5037, Purina Mills, St Louis, MO) twice daily. Consumption was monitored and adjustments were made as necessary depending on sex, age, and weight so that animals received sufficient food without creating excessive food waste. SIV-infected Rhesus macaques were housed in adjoining individual primate cages allowing social interactions and participation in enrichment activities, under controlled conditions of humidity, temperature and light (12-hour light/12-hour dark cycles). Appropriate procedures were performed to ensure that potential distress, pain, discomfort and/or injury was limited to that unavoidable in the conduct of the research plan. All enclosures and animal rooms were cleaned daily with water and sanitized at least once every two weeks. The sedatives Ketamine (10 mg/kg) and/or Telazol (4 mg/kg) were applied as necessary for blood collections and analgesics were administered as appropriate by the veterinary medical staff. If an animal had developed a disease state that was not clinically manageable, the animal would have been euthanized in accordance with the recommendations of the Panel on Euthanasia of the American Veterinary Medical Association (AVMA). No animals were euthanized before the latest time points used in the present study. These animals were included in parallel projects and were euthanized using sodium pentobarbital. Euthanasia was ensured by exsanguination and bilateral pneumothorax, consistent with the recommendations of the AVMA Guidelines on Euthanasia (June 2007).

### Animal study design

**Fig 1:**
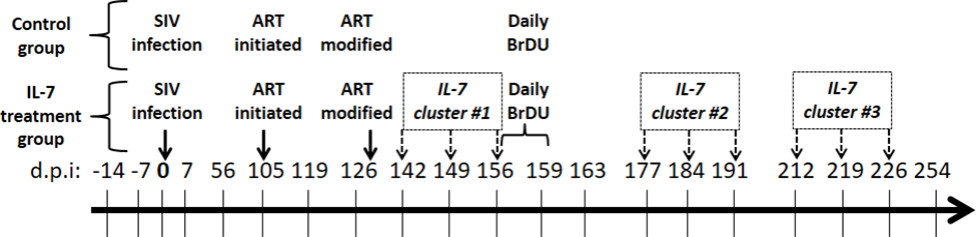
Study design for the SIV infection, antiretroviral treatment and IL-7 therapy administration of Rhesus macaques. Animals were infected with SIV at day 0, and antiretroviral (ART) was initiated 105 days post-infection (d.p.i), with a dose reduction 128 d.p.i. Animals who were administered rsIL-7-gly received 3 clusters of rsIL-7gly administered s.c. weekly for 3 weeks followed by 2 weeks between each cluster. All animals were administered BrDu daily for 4 days, starting 149 d.p.i. Each vertical line corresponds to date on which a collected plasma sample was utilized for the quantification of sCD127 in this study.

A schematic of the experimental design is shown in Fig 1. A total of 9 Rhesus macaques were analyzed in this study, many of whom were the subject of a previous study by Leone A. (now Steele, AK) et al.[34] All animals were infected with SIVmac239, i.v. using 5 ng equivalents of SIV p27 (1.03105 infectious centers) (day 0), treated with ART (105 days post-infection (d.p.i.)). All animals were started on 9-R-2-phosphonomethoxypropyl adenine (PMPA) (30mg/kg) and emtricitabine (FTC) (50mg/kg) for 4 weeks. The ART was then reduced to a maintenance dose (PMPA 20mg/kg; FTC 50mg/kg).

Following SIV infections, animals were divided into a control group (n = 4) or IL-7-treated group (n = 5). The IL-7 treatment group was administered 3 clusters of rsIL-7gly (30mg/kg, Cytheris) administered s.c. weekly for 3 weeks followed by 2 weeks between each cluster. The control group consisted of animals RM23092, RM23699, RM23788 (previously RM2-06 in Leone et al., 2010) and RM23892. The IL-7-treated animals were RM22657 (previously RM2-05), RM23201 (previously RM2-07), RM23686, RM23750 and RM24090.

### T-cell phenotyping and related immunoassays

Cells were phenotyped by flow cytometry as described in the previously published study[34]. The present study referred specifically to naïve, central and effector memory cell subsets identified as follows: naïve (T_N_, CD28^int^CD95^low^CCR7^int^CCR5^-^), central memory (T_CM_, CD95^hi^CD28^hi^CCR7^+^CCR5^-^), transitional effector memory (T_TrM_, CD28^hi^CCR5^+^ and/or CCR7^-^) and fully differentiated effector memory (T_EM_, CD95^hi^CD28^-^CCR7^-^CCR5^dim+^). T-cell activation was determined by assessing Ki67 expression by flow cytometry. Lastly, the retention of T-cells was determined by enumerating the number of BrdU^+^ cells following in vivo labeling with BrdU, as described previously[35].

### Quantification of plasma sCD127 concentrations

Plasma samples used in this study corresponded to specific time points prior to SIV infection, after ART initiation, and during the period of IL-7 administration. All plasma samples were treated with Triton-X (0.1%) at 37°C for 1 hour to neutralize viral particles, and then diluted to 1/10 in PBSBN buffer (PBS + 1% BSA+ 0.05% NaN_3_).

To quantify the concentration of sCD127 in Rhesus macaque plasma, a CD127-specific antibody-microfluorosphere MagPix assay was adapted from the original human CD127-specific Luminex assay[36] that was found to cross-react with simian CD127. The goat anti-human CD127 capture antibody (R&D Systems) was coupled to MagPix beads (Luminex, MC10025-01) using the XMAP antibody coupling kit (Luminex) and following the manufacturer’s instructions. Briefly, 1.7x10^7^ microspheres were coupled to 80 μg of capture antibody and stored at 1.x10^7^ beads/ml in the dark at 4°C until needed. A Bio-Plex Pro 96-well flat bottom plate (Bio-Rad, 171–025001) was pre-wet with 200 μl/well of PBST (PBS + 0.05% Tween 20 + 0.05% NaN_3_). Following a soak-aspirate wash on the automated Bio-Plex Pro II wash station, 50 μl/well of PBST + 50 μl/well of MagPix-coupled capture antibody (6x10^4^ beads/ml) were added. A soak-aspirate wash was repeated, followed by 3 dispense-soak-aspirate cycle washes of 200 μl/well of PBST. Then, 50 μl/well of sample/well was added and plate was shaken (800 rpm) for 2 hours at room temperature. The wash steps above were repeated. The detection antibody was added (50 μl/well, biotinylated mouse anti-human CD127, 20.4 μl/ml of PBSBN, BD Pharmingen) and the plate was shaken (800 rpm) for 1 hour at room temperature. Following repeated washing, 50 μl/well of streptavidin R-phycoerythrin conjugate (1/500 in PBSBN, Invitrogen) was added and the plate was shaken (800 rpm) for 10 minutes at room temperature. The plate was washed and 125 μ/well of 2% paraformaldehyde was added, followed by shaking the plate at 1000 rpm for 60 seconds. Bi-fluorescent complexes were then detected with the Bio-Plex reader and Bio-Plex Manager 6.0 software (BioRad). The concentration of sCD127 in plasma samples was determined by extrapolating their fluorescent readings from a standard curve of recombinant sCD127 Fc-chimeric protein (1–500 ng/ml) prepared in a 1/10 dilution of sCD127 negative plasma. Positive controls for this assay included 1:1 and 1/10 dilutions of culture supernatant collected from WI-26VA4 cells, known to secrete sCD127[37].

### Data analyses

All data sets were graphed using GraphPad Prism software 5.0 (GraphPad). A paired one-way Student’s *t*-test or ANOVA followed by a Kruskal-Wallis non-parametric post-test were used as appropriate to detect statistically significant changes in plasma sCD127 concentration. Data pertaining to viral load, T-cell numbers, activation state (Ki67+/-), and survival were extracted from the study files associated with the Leone A et al. study[34], and its associated PhD thesis. The extracted data was used in correlation studies with the measured sCD127 concentrations. The Spearman rank correlation test was used to determine whether there was a significant association between two variables. Multiple regression coefficient analyses were used to detect significant associations between sCD127 concentrations and T-cell parameters over time using XLSTAT (Addinsoft, New York, NY, USA). All raw data can be found on the Dryad online filesharing platform (DOI: https://doi.org/10.5061/dryad.72c40/1).

## Results

### Plasma sCD127 concentrations are not altered by SIV infection or ART

**Fig 2:**
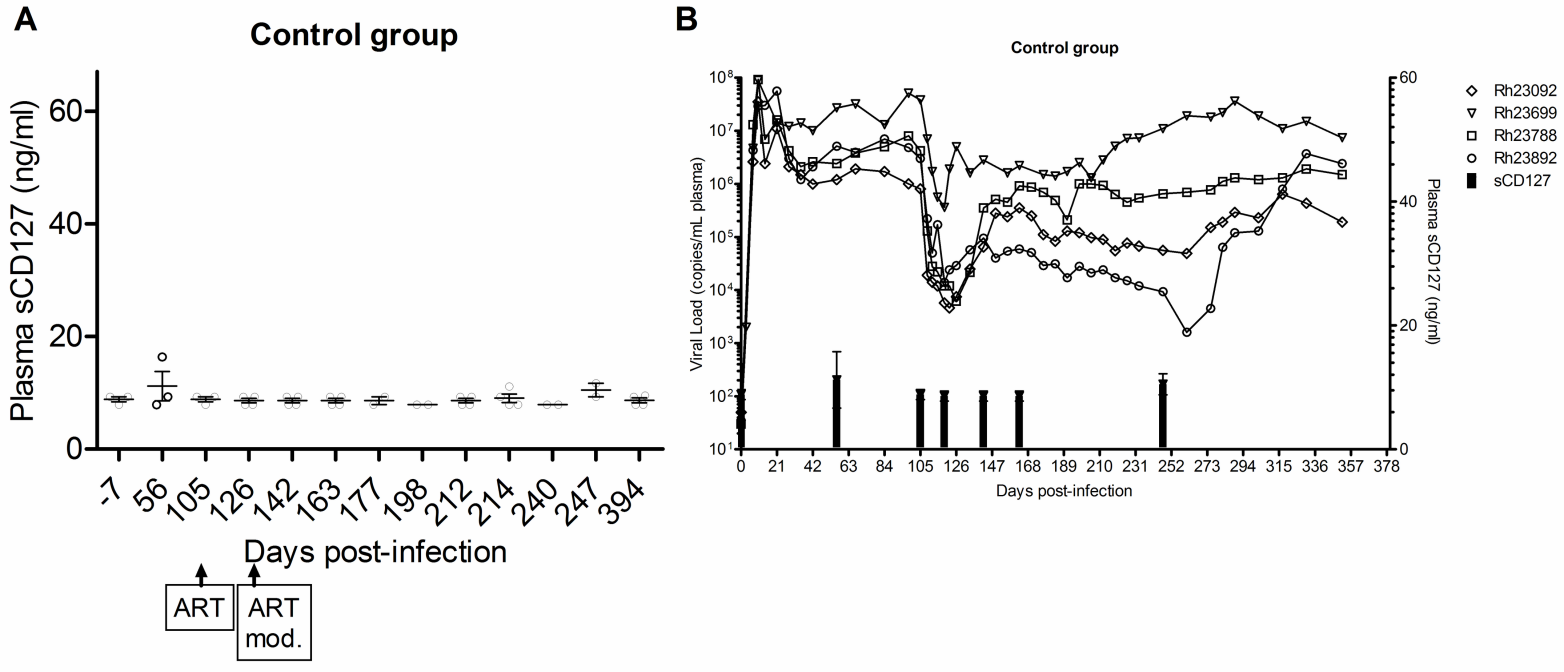
Plasma sCD127 concentrations are unaffected by SIV infection or antiretroviral therapy in Rhesus macaques. The concentration of sCD127 in plasma samples of Rhesus macaques prior to SIV infection, after infection, following ART initiation (>105 d.p.i.) was determined using a CD127 microbead immunoassay. The minimum detection limit of the immunoassay is 5 ng/ml. (A) Plasma concentrations of sCD127 did not change following SIV infection or after ART (paired Student’s *t*-test, n = 4) in the control group. (B) The concentration of plasma sCD127 (mean, +/- SD) is shown relative to viral loads for each animal in the control group over time (0–380 d.p.i.). Plasma samples for RM23892 (day -7 until day 105 post-infection) could not be located for the quantification of sCD127 in this retrospective study.

To determine the effect of SIV infection on plasma sCD127 concentrations, an immunobead assay was used to quantify the concentration of this soluble receptor in plasma collected from Rhesus macaques prior to, and immediately following primary SIV infection. Pre-infection, the concentration of plasma sCD127 varied from undetectable to 25.79 ng/ml (±7.4 S.D., n = 9). The concentration of plasma sCD127 was not significantly altered at 56 or 105 d.p.i. compared to day 0 (analysed by two-tailed Student’s *t*-tests), suggesting that early SIV infection did not significantly alter sCD127 expression (Fig 2A).

ART was initiated 105 d.p.i and did not significantly alter plasma sCD127 concentrations at 126 and 142 d.p.i. in the control group (n = 4, Fig 2A). The plasma concentrations of sCD127 remained unchanged long-term (i.e. up to 394 d.p.i., ANOVA and Kruskal-Wallis post-test) in the control macaques that did not go on to receive IL-7 therapy (Fig 2A). This result was independent of viral load, although all animals in the control group failed to suppress SIV viral loads with ART (Fig 2B).

### Soluble CD127 increases with IL-7 treatment in ART-treated SIV^+^ Rhesus macaques

**Fig 3:**
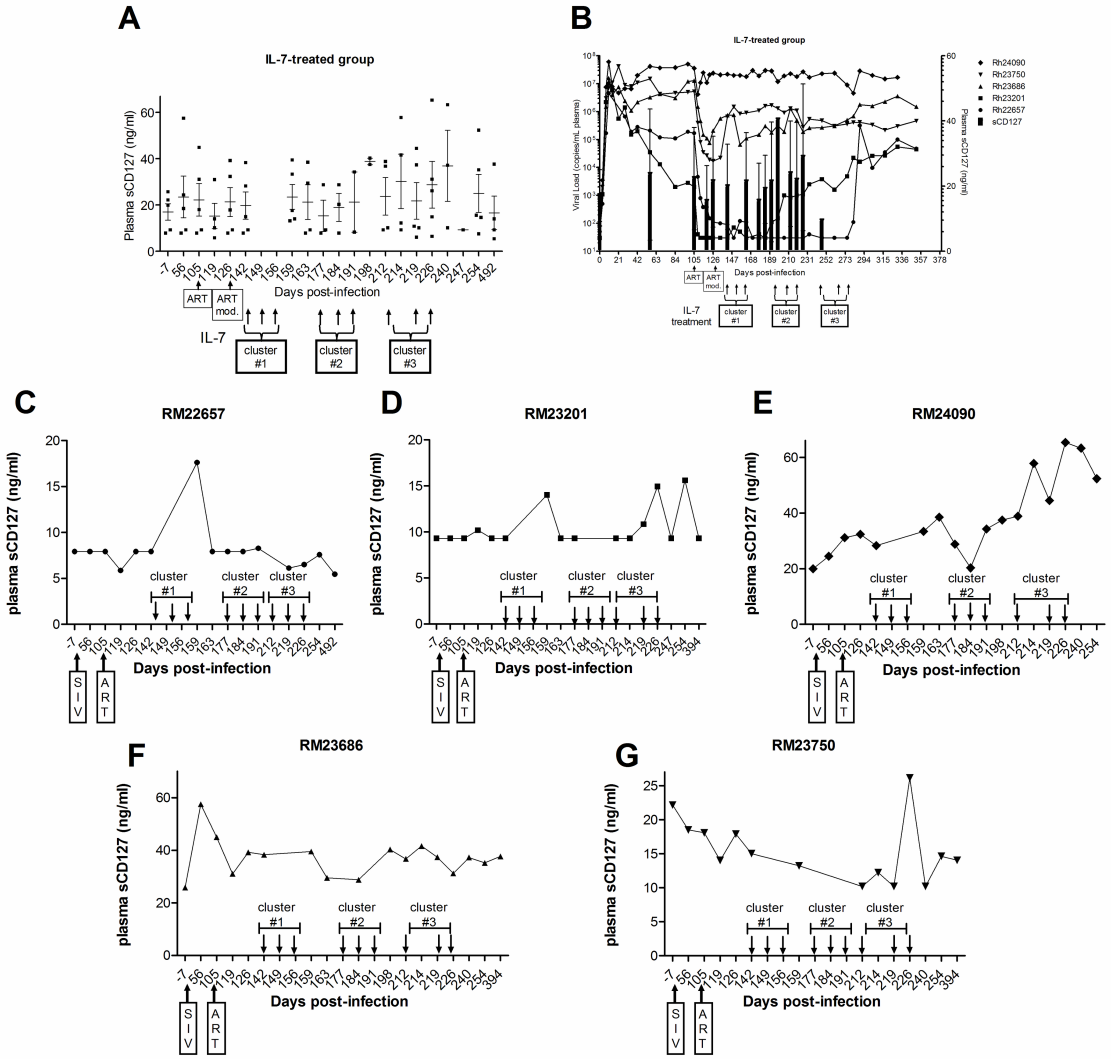
Plasma sCD127 concentrations increased and persisted after IL-7 administration to ART-treated SIV-infected Rhesus macaques. The concentration of sCD127 in plasma samples of Rhesus macaques prior to SIV infection, after infection, following ART initiation (>105 d.p.i.) and after each administration of IL-7 (started at 142 d.p.i.) was quantified by a CD127 microbead immunoassay (min. detection limit: 5 ng/ml). (A) Plasma sCD127 concentrations transiently increased in all SIV-infected, ART-treated animals that were administered rsIL-7-gly (n = 5). In some cases, these increases persisted for 3–5 weeks. (B) The concentration of plasma sCD127 (mean, +/- SD) is shown relative to viral loads for each animal in the IL-7-treated group over time (0–380 d.p.i.). (C-G) Changes in sCD127 concentrations also varied by IL-7 cluster, depending on the individual. In the first cluster of IL-7 administration, 3 of 5 animals (C, D and E) demonstrated a sharp increase in plasma sCD127 concentrations before the next cluster. The second cluster of IL-7 treatment increased sCD127 release in 2 animals (E and G). Plasma sCD127 concentrations increased in the third cluster in 3 of 5 animals (D, E and G). Transient increases in plasma sCD127 concentrations persisted for as long as 3–5 weeks following the last administration of IL-7 in a given cluster. Lastly, the magnitude and frequency of observed increases in sCD127 concentrations was most pronounced in animals that either (C) failed to suppress the virus with ART or (D, F and G) lost virological control, compared to (C) the virologically suppressed animal. The clustered administration of IL-7 is indicated on the x-axis by groups of three arrows labeled as clusters #1–3.

Given that IL-7 has been shown to induce the release of CD127 from human T-cells, the effect of IL-7 therapy on the concentration of plasma sCD127 was investigated in the SIV-infected Rhesus macaques. Administration of IL-7 did not lead to consistent significant increases in the mean sCD127 concentrations at any time point (p > 0.05 by ANOVA, Fig 3A). Monitoring of plasma SIV viral loads throughout the IL-7 treatment periods found that RM22657 maintained viral control, and RM24090 failed to suppress, while RM23201 (191 d.p.i), RM23686 (134 d.p.i) and RM23750 (134 d.p.i) lost viral control (at the indicated dates post-infection) (Fig 3B). Based on the responses of individual animals, it appeared that the degree of viral suppression may have affected the changes in sCD127 concentrations in response to IL-7 therapy. For example, RM22657 (Fig 3C), who transiently increased sCD127 in only one cluster, had an undetectable viral load (< 50 copies/ml) as of 170 d.p.i. on ART. Animals who were suppressed but lost viral control when the ART dose was changed (RM23201 [Fig 3D], 23686 [Fig 3F], and RM23750 [Fig 3G]) had increased sCD127 concentrations in either cluster 2 or 3 when the viral load had fully rebounded. Finally, RM24090, who transiently increased sCD127 in response to all three IL-7 clusters, failed to suppress viral load on ART (22 x 10^6^ copies/ml, 142 d.p.i.) at any time point (Fig 3E). In addition, the magnitude of the sCD127 burst in RM24090 increased with each treatment cluster. Thus, in individual animals, the lack of viral control tended to increase the magnitude and frequency of sCD127 increases in response to IL-7 therapy.

While there was no statistically significant increase in sCD127 concentrations in a group analysis (Fig 3A), each of the five IL-7-treated macaques responded to at least one cluster of IL-7 doses with transiently increased concentrations of sCD127. RM 24090 (Fig 3E) was the most responsive to IL-7 administration and showed transient increases in sCD127 following all three IL-7 clusters. RM23201 (Fig 3D) transiently increased sCD127 concentrations following two of the three IL-7 clusters, and RM22657 (Fig 3C), RM23686 (Fig 3F), and RM23750 (Fig 3G) transiently increased sCD127 following one of the three IL-7 clusters. In some cases, the transient increase in plasma sCD127 concentrations persisted for as long as 3–5 weeks following the last administration of IL-7 in a given cluster. Therefore, IL-7 therapy appeared to induce transient increases in plasma sCD127 concentrations in SIV-infected, ART-treated Rhesus macaques.

### Plasma sCD127 correlates with IL-7effects on CD8^+^ T-cell numbers in ART-treated SIV^+^ Rhesus macaques

In the control animals, there was no significant correlation between the sCD127 concentration and the absolute CD4^+^ or CD8^+^ T-cell numbers (Spearman correlation analysis) in early SIV infection (before 105 d.p.i.). There were also no significant associations between sCD127 concentrations and the absolute numbers of CD4^+^ or CD8^+^ T-cells from 142–247 d.p.i. when time was taken into account in a multiple regression coefficient analysis. Therefore, in the context of ART alone, the concentrations of sCD127 in the plasma did not appear to be correlated with the absolute number of total CD4^+^ or CD8^+^ T-cells.

**Fig 4:**
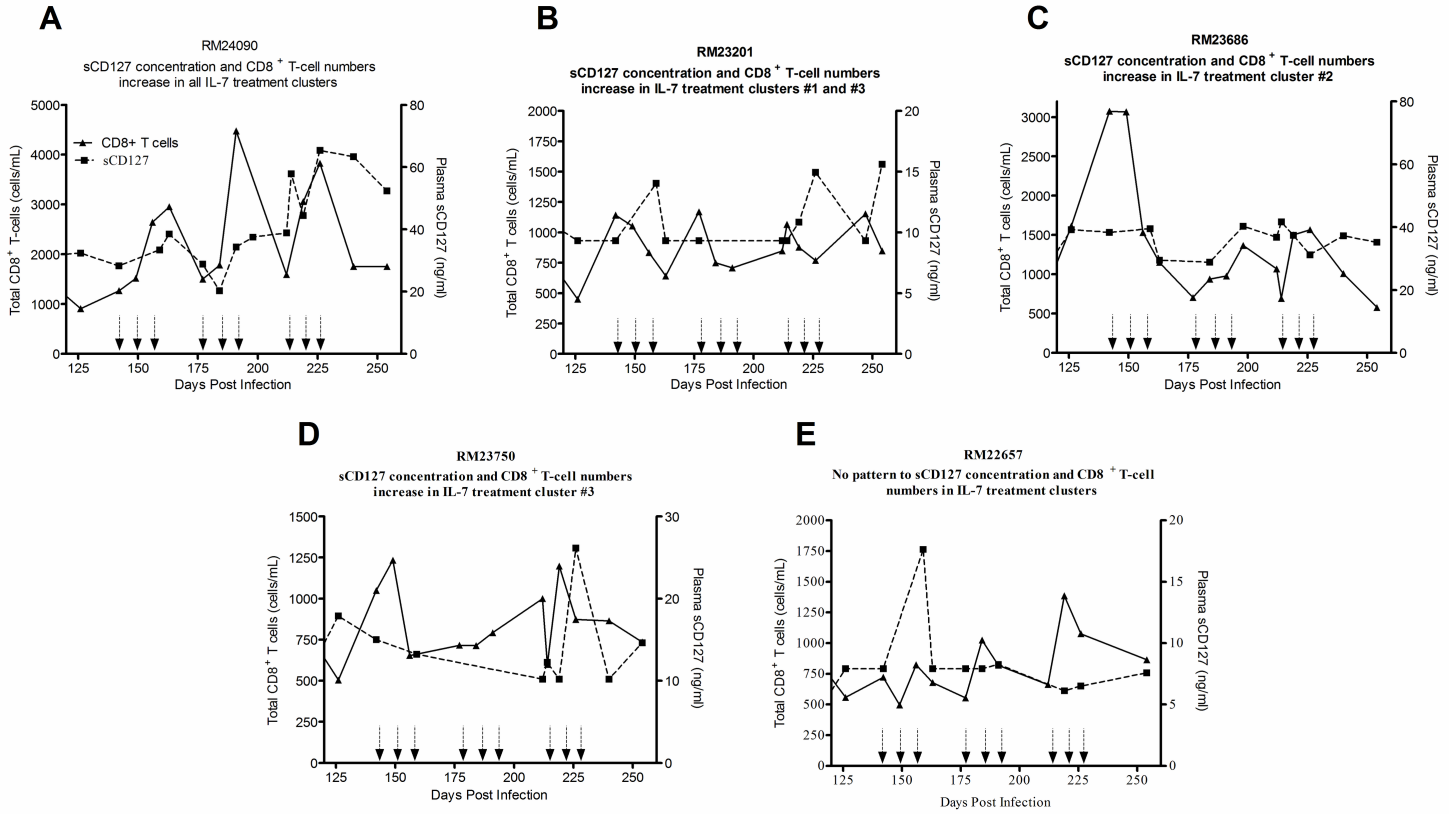
Increased plasma sCD127 concentrations correlated with IL-7-induced increases in CD8^+^ T-cells in SIV-infected Rhesus macaques. (A-D) Representative graphs of IL-7-treated animals whose concentration of plasma sCD127 increased with changes in absolute CD8^+^ T-cell numbers in at least one IL-7 cluster during the cytokine treatment time period. (E) In the animal that maintained viral control, plasma sCD127 concentrations did not appear to associate with changes CD8^+^ T-cell numbers. Total number of IL-7-treated animals in which data of absolute T-cells was available: n = 5. The clustered administration of IL-7 is indicated on the x-axis by groups of three arrows.

**Fig 5:**
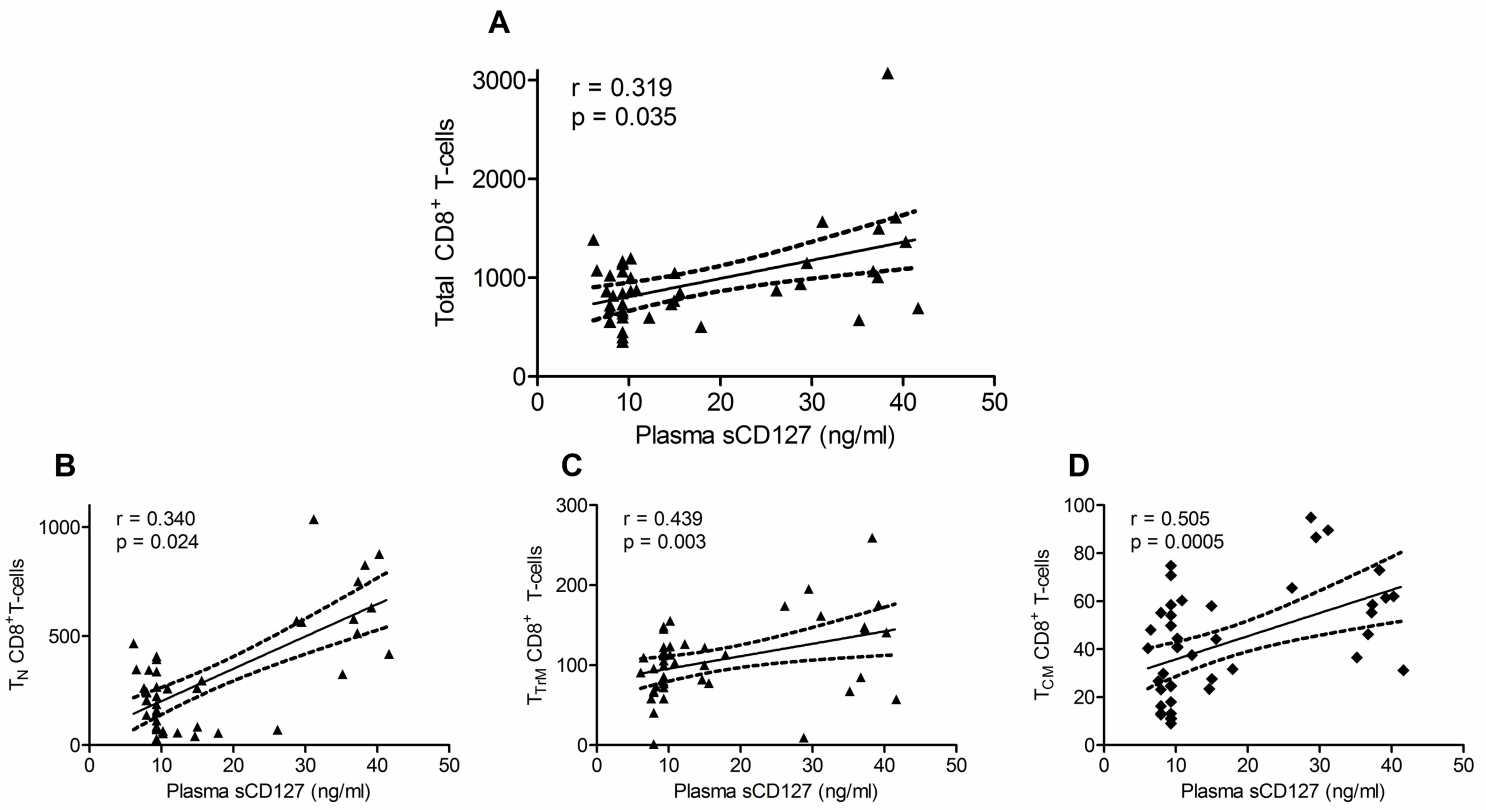
Increased plasma sCD127 concentrations correlated with IL-7-induced increases in CD8^+^ T-cell subsets in SIV-infected Rhesus macaques. All CD8^+^ T-cell subsets with statistically significant correlations with sCD127 are graphed here. Increasing concentrations of plasma sCD127 following IL-7 administration were positively correlated with increases in (A) total, (B) naïve T_N_, (C) transitional effector memory T_TrM_ and (D) central memory T_CM_ CD8^+^ T-cell subsets in SIV-infected, ART-treated Rhesus macaques (Spearman correlation analysis, r- and p-values are indicated on graphs).

In contrast, in the macaques treated with IL-7, there were distinctive features in the changes in sCD127 concentrations and absolute CD8^+^ T-cell numbers during the IL-7 treatment period. Taking a more granular approach, and again focusing on the responses of individual animals, RM24090 (Fig 4A) had the most apparent association between sCD127 concentrations and total CD8^+^ T-cell numbers. In this animal, both the plasma sCD127 concentration and T-cell number increased in response to each cluster of IL-7. The animals that lost control of their viral load (RM23201 [Fig 4B], RM23686 [Fig 4C], and RM23750 [Fig 4D]) also had some degree of overlap between increased plasma sCD127 concentrations and increased total CD8^+^ T-cells in response to specific IL-7 clusters. In contrast, there was no visible relationship between sCD127 and CD8^+^ T cell numbers in RM22657 who maintained viral suppression. In this animal, the single increase of sCD127 in cluster 1 was not coupled by an increase in the absolute T-cell numbers (Fig 4E). In contrast, assessment of sCD127 plasma levels and total CD4^+^ T-cell numbers during IL-7 therapy did not exhibit a significant correlation (Spearman rank correlation test, r = 0.345, p = 0.066). There was an overall strong, positive correlation between sCD127 concentrations and total CD8^+^ T-cell numbers (Fig 5A, Spearman, r = 0.3193, p = 0.035) during the IL-7 treatment period (142–254 d.p.i). Among the CD8^+^ T-cell subsets, this was found to most significant in the T_N_ (r = 0.340, p = 0.024, Fig 5B), T_TRM_ (r = 0.439, p = 0.003 Fig 5C) and T_CM_ (r = 0.505, p = 0.035, Fig 5D) cells. There was no significant correlation between sCD127 and the absolute numbers of CD3^+^, CD4^+^ or T_EM_ CD8^+^ T-cells during the IL-7 treatment period. When taking time into account, increased sCD127 concentrations significantly correlated with increasing absolute CD8^+^ T-cell numbers over the course of the IL-7 treatment (multiple regression coefficient analysis, r = 0.490, p = 0.004). This was particularly evident in the same CD8^+^ T-cell subsets as in the previous analysis: T_N_ (r = 0.708, p < 0.001), T_TRM_ (r = 0.421, p = 0.018) and T_CM_ (r = 0.519, p = 0.002).

### Plasma sCD127 concentrations associate with IL-7-mediated activation of T-cells in ART-treated SIV^+^ Rhesus macaques

In this cohort of macaques, T-cell activation (% Ki67^+^) was significantly increased in response to IL-7 and mirrored the observed increases in the absolute numbers of T-cells[34]. To further characterize the role of sCD127 concentrations in T-cell activation, the correlation between changes in plasma sCD127 concentrations and the number of Ki67^+^ T-cells following the initiation of IL-7 treatment was evaluated. Due to the retrospective nature of the study, this data was only available for 4 of the 5 animals treated with IL-7, as analyzed in the T_EM_ and T_CM_ subsets.

**Fig 6:**
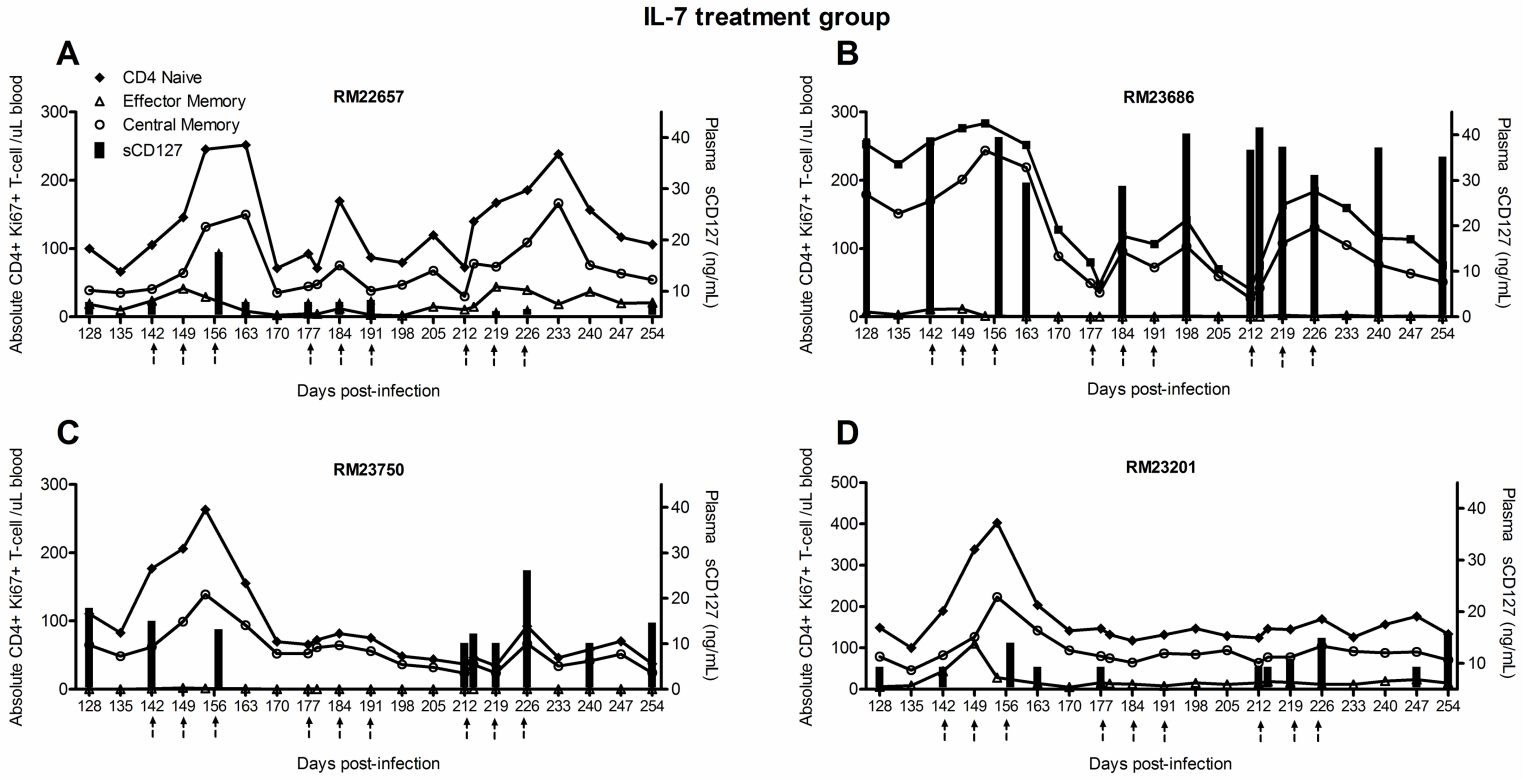
Plasma sCD127 concentrations associated with IL-7-induced activation of CD4^+^ T-cells in SIV-infected, ART-treated Rhesus macaques. These effects were observed frequently among animals in this study, yet varied by IL-7 treatment cluster (depicted by arrows). (A-D) Increases of sCD127 paralleled the changes in Ki67^+^CD4^+^ T_N_ and T_CM_ cell numbers in 3 out of 4 animals evaluated, specifically (A) RM22657, (B) RM23686 and (C) RM23750.

**Fig 7:**
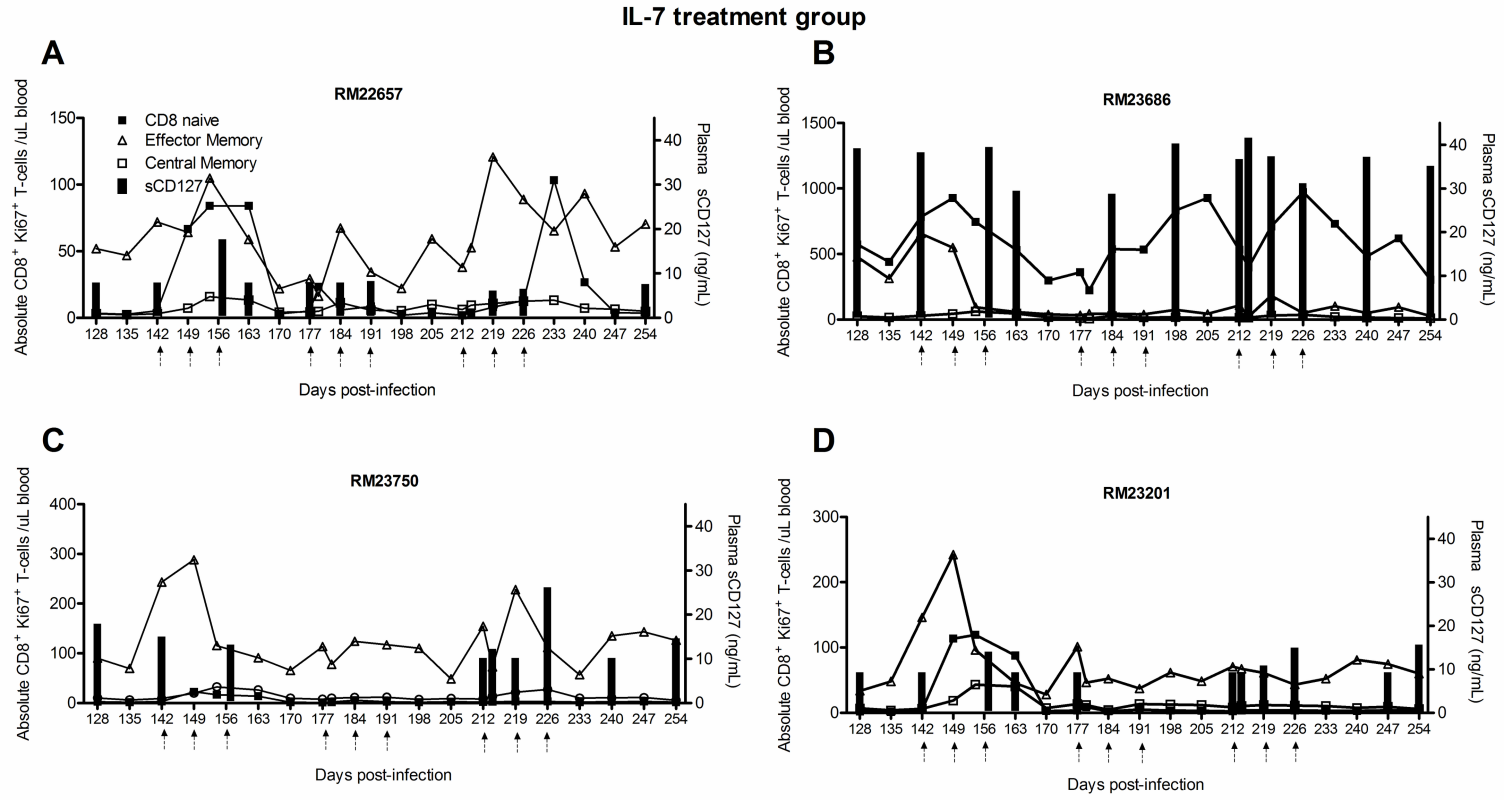
Plasma sCD127 concentrations associated with IL-7-induced activation of CD4^+^ T-cells in SIV-infected, ART-treated Rhesus macaques. The concentration of plasma sCD127 increased as the number of activated CD8^+^ T_EM_ cells increased in all 4 animals treated with IL-7. Total number of IL-7-treated animals in which T-cell activation data (Ki67 assay) was available: n = 4 (data for the 5^th^ animal in this group is not available for this parameter of the study). The clustered administration of IL-7 is indicated on the x-axis by groups of three arrows.

Given the individual variation in sCD127 concentrations and the percentage of activated memory CD4^+^ and CD8^+^ T-cell subsets, there was no detectable significant correlation between sCD127 and the percentage of Ki67^+^ CD4^+^ T-cells (Fig 6A–6D) or CD8^+^ T-cells (Fig 7A–7D). However, on an individual concentration, in 3 of the 4 animals for whom this data was available, sCD127 concentrations increased in parallel with changes in Ki67^+^CD4^+^ T_N_ and T_CM_ cell numbers: RM22657 in the first cluster of IL-7 treatment (156 d.p.i., Fig 6A); RM23686 in the second cluster increased sCD127 concentrations between 184–212 d.p.i., prior to an increase in the number of these T-cell subsets (Fig 6C); and RM23750 showed an increase of plasma sCD127 in cluster 3 (212–226 d.p.i.) in tandem with increasing Ki67+ T_N_ and T_CM_ CD4^+^ cells (Fig 6D).

The concentration of sCD127 also increased in parallel with the number of activated CD8^+^ T_EM_ cells in all 4 animals for whom this data was available (Fig 7): at 156 d.p.i., plasma sCD127 concentrations rebounded in RM22657 (Fig 7A) and RM23201 (Fig 7D) when Ki67^+^CD8^+^ T_EM_ cell numbers increased; increasing sCD127 concentrations in RM23686 during 181–212 d.p.i. occurred before a subsequent burst in activated CD8^+^ T_EM_ cell numbers 219 d.p.i. (Fig 7B); and lastly, sCD127 concentrations increased in RM23750 between 212–226 d.p.i. when cell activation increased 226 d.p.i. (Fig 7C).

### Plasma sCD127 may contribute to IL-7-mediated retention of T-cells

**Fig 8:**
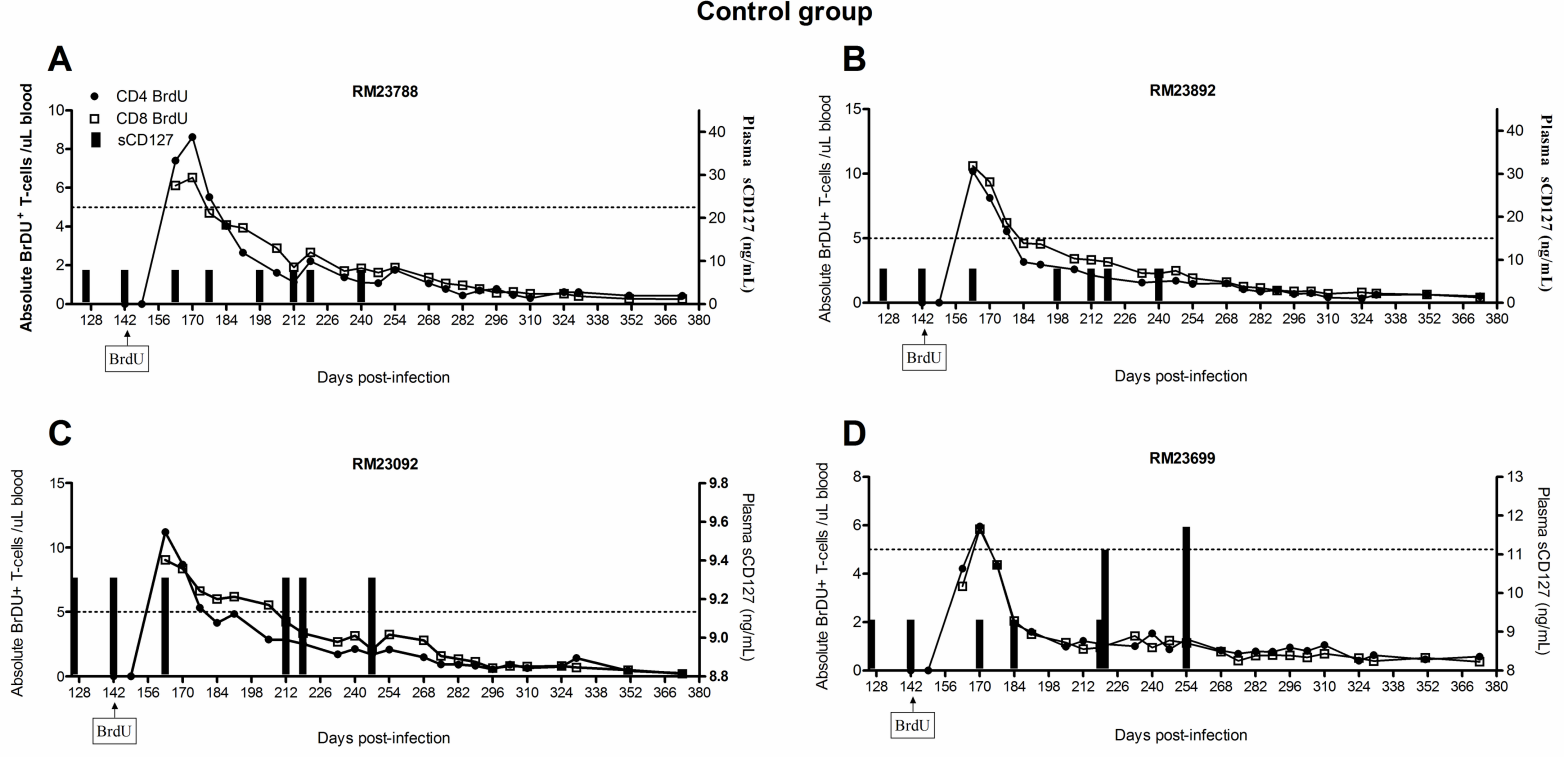
IL-7 therapy-mediated effects on T-cell survival is associated with increases in plasma sCD127 concentrations. The concentration of plasma sCD127 was compared to T-cell survival, as assessed by BrDU uptake. Starting at 142 d.p.i, animals received 4 daily injections of BrDU. The number of circulating BrDU^+^ CD4^+^ and CD8^+^ T-cells was determined every 7 days until 373 d.p.i. (A) The survival profiles of T-cells in each of the SIV-infected, ART-treated Rhesus macaques in the control group are shown.

The survival of the T-cells proliferating in response to IL-7 therapy *in vivo* was assessed in this cohort of macaques using BrdU labeling. The animals received BrDU for 4 days beginning seven days after the first dose of IL-7 therapy (i.e. 149 d.p.i). Retention (i.e. survival) of proliferating cells was defined as detection of BrdU^+^ cells over time, reflecting an increased half-life for labeled cells. IL-7 therapy increased the retention of memory T-cells in SIV^+^ ART-treated Rhesus macaques, indicating a survival benefit in addition to a proliferative effect[34]. In this study, we hypothesized that the release of sCD127 may have been related to the cell survival effects observed with IL-7 therapy. In control animals, the number of BrDU^+^ memory CD4^+^ or CD8^+^ T-cells were less than an arbitrary yet common crossing point of 5 cells/μl of blood within ≈ 2 weeks of BrdU administration (CD4 memory mean 16.5 ±9 days post-BrdU; CD8 memory mean 23.5 ±19 days post-BrdU, Fig 8). However, given that the concentration of sCD127 was low and not altered in the control animals, it was not possible to evaluate a relationship between sCD127 concentrations and memory T cell survival in this population. Therefore, our analyses focused exclusively on the IL-7-treated group.

**Fig 9:**
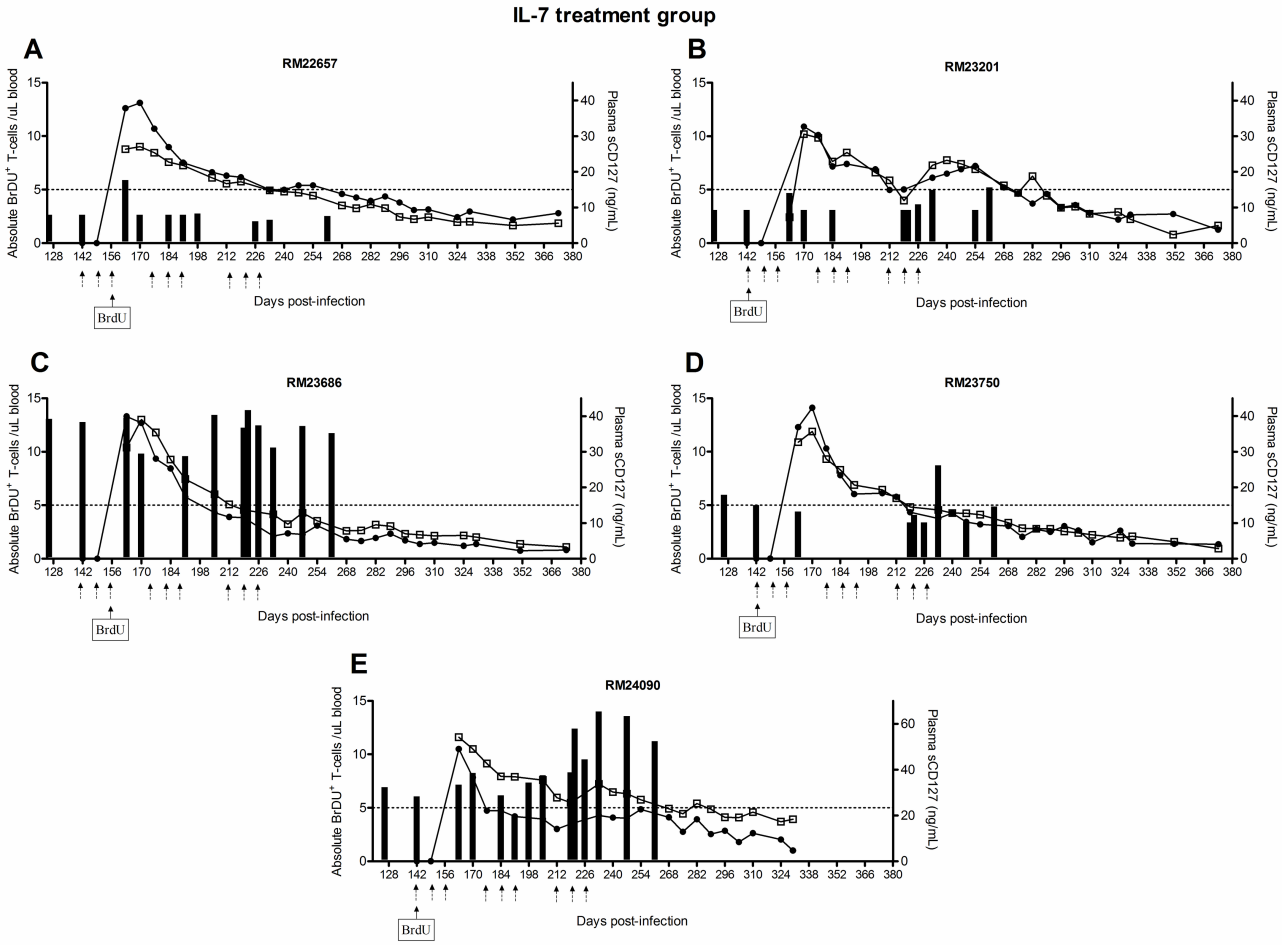
IL-7 therapy-mediated effects on T-cell survival is associated with increases in plasma sCD127 concentrations. T-cell survival graphs for all animals in the IL-7 treatment group. In all plots, an arbitrary, yet common crossing point of 5 BrDU^+^ cells/μl of blood is indicated by a dotted line. The clustered administration of IL-7 is indicated on the x-axis by groups of three arrows and the first day of the BrDU injections is also indicated.

When analyzing individual responses to IL-7 treatment, there was some evidence that sCD127 concentrations were associated with the persistence of BrDU-labeled cells. Coincident with the increase in sCD127 concentrations in IL-7-treated Rhesus macaques compared to those treated with ART only (Fig 2A compared to 3A), a significant survival benefit was observed in all IL-7-treated animals, as shown by the delayed loss of BrDU^+^ cells (Fig 9). In contrast to the ART-treated control group, animals receiving ART + IL-7 treatment retained at least 5 BrDU^+^ cells/μl of blood as late as 254 d.p.i. The degree of viral suppression also appeared to influence sCD127 concentrations in relation to when the number of BrDU^+^ cells fell below the 5 cell/uL threshold. Among the four animals who either lost viral control or failed to suppress viremia on ART and had high concentrations of sCD127, three recorded < 5 BrDU^+^ CD8^+^ cells/uL by ≈ 212 d.p.i.: RM23201 (Fig 9B), RM23686 (Fig 9C) and RM23750 (Fig 9D). This was considerably sooner than the animal with full virological control (RM22657, Fig 9A) whose CD8^+^ T-cell numbers were below this minimum after ≈ 240+ d.p.i. This animal had intermediate concentrations of sCD127 that did not fluctuate over time.

## Discussion

Therapeutic administration of IL-7 has the potential to restore T-cell numbers and increase cell survival[7, 8, 15]. The present study has found that plasma sCD127 concentrations parallel these outcomes in SIV-infected, ART-treated Rhesus macaques administered IL-7. Repeated injections of IL-7 resulted in transiently increased concentrations of sCD127 that were affected by the degree of viral suppression in each animal. These increases in sCD127 concentrations associated with IL-7-induced increases in the absolute numbers and number of activated CD8^+^ and CD4^+^ T-cells. The increases in plasma sCD127 were also associated with IL-7-enhanced T-cell survival, while having an inverse relationship with T-cell retention over time following IL-7 treatment. These data show for the first time that a parallel increase in plasma sCD127 concentrations with IL-7 therapy is a biomarker for increased numbers and survival of T-cells in the ART-treated SIV infection model.

In IL-7-treated animals, poor viral control was also associated with a greater increase in sCD127 as well as how often a given animal experienced an increase in sCD127 in response to an IL-7 cluster. Carini et al. were the first to show that viral control was associated with sCD127 release. They demonstrated that PBMC from untreated HIV^+^ individuals lacking CTL responses with IL-7 stimulation released more sCD127 *in vitro* than those of individuals with detectable CTL responses[24]. Likewise, we have previously shown that increased plasma sCD127 concentrations in untreated HIV infection positively correlated with plasma IL-7 concentrations, linking chronic viral infection and IL-7 with sCD127 release[23]. Other reports of sCD127 concentrations in HIV infection have found either decreased concentrations or similar concentrations, when compared to healthy individuals[38, 39]. Janot-Sardet et al., evaluated IL-7 therapy in HIV infection yet found not change in sCD127 concentration. It should be noted that those study subjects were ART-treated and aviremic (>12 months) and were administered recombinant glycosylated *human* IL-7 at a 3x lower dose than our study, and this was administered weekly for only 3 weeks, compared to our 3 clusters[40]. Therefore, multiple inherent differences in the study design make comparison of the present data to that study problematic. What is notable of the data reported herein is that increases in sCD127 concentrations and its association with increased effects on T-cells were more pronounced when SIV infection was not controlled. There is increasing evidence to suggest that sCD127 concentrations may have a role in chronic disease. The CD127 gene has distinct genetic features that suggest it may be a biomarker for the progression of chronic immune-based diseases, as initially discovered in multiple sclerosis[41–43]. In addition to this, we and others have reported associations of sCD127 concentrations with disease in HIV infection[18, 22–25], Mycobacterium tuberculosis infection[44], stem cell transplantation[45, 46], T-cell acute lymphoblastic leukemia[47], Type 1 diabetes (T1DM)[48] and rheumatoid arthritis[49]. In addition, the presence of sCD127 has been associated with enhanced IL-7-mediated homeostatic proliferation and exacerbation of existing T-cell-derived autoimmunity[27]. Similarly, IL-7-mediated anti-tumour activity of T-cells was significantly enhanced in the presence of sCD127[50].

As the Leone et al. study, and that of others, have shown, IL-7 therapy in the context of ART-treated SIV/HIV results in immunorestorative effects on T-cells[7, 8, 33]. This is potentially an important gain, given that viral suppression does not result in complete immune reconstitution of the T-cell compartment. IL-7 therapy can improve CTL activity (e.g. ↑IFN-γ), viral clearance and mitigate associated liver pathology in a murine model of chronic LCMV infection[51], a system sharing some similarities to HIV and HCV infection. In this study, we found associations suggesting that sCD127 may be contributing to the effects of IL-7 therapy on T-cells and therefore, perhaps sCD127 is a biomarker for immunorestorative effects. The challenge for the eradication of HIV infection is the elimination of the latent viral reservoir. Recent *in vitro* findings have demonstrated that IL-7 may be reactivating the HIV reservoir in memory CD4^+^ T-cells in otherwise virally-controlled settings[52]. This follows a “kick and kill” HIV cure strategy whereby inducing viral replication in the reservoir cells enables antiviral drug elimination of these final sources of virus[53]. Such effects with IL-7 therapy were observed in the first HIV trials with this cytokine, as transient viral blips were recorded following cytokine administration[7, 8]. Whether sCD127 has a role in the immunorestorative and viral reservoir reactivation effects of IL-7 therapy through its enhancement of T-cell numbers is not known.

While increases in plasma sCD127 concentrations correlated with, or were coincidental with several IL-7-induced outcomes measured in this model, whether sCD127 concentrations directly contributed to the outcomes achieved is not known[34]. Despite being discovered more than 20 years ago, the role of sCD127 in mediating IL-7 activity has not been well characterized[37], and hence the extent to which it contributes to disease remains unclear. In our early research, experiments using either native or recombinant sCD127 demonstrated a reduction of IL-7-mediated CD8^+^ T-cell signaling and proliferation *in vitro*[23]. *In vivo* experiments reported that the release of sCD127 at the onset of Type 1 diabetes was associated with inhibiting IL-7-mediated signaling and cell proliferation[54]. This was thought to be mediated in part by increased glycosylation of sCD127 during hyperglycemia, impairing the ability of sCD127 to inhibit IL-7 activity[54]. However, our more recent studies demonstrate that pre-incubation of sCD127 with IL-7 increased cytokine activity on CD8^+^ T-cells, and enhanced their cellular function[26]. Other studies have also shown a similar augmentation of T-cell activity in tumour models and in the context of autoimmunity[27, 50]. Taken together, these studies confirm previous suggestions that sCD127 may act as a either a decoy receptor or extending cytokine action by prolonging the half-life of its ligand[55]. The data presented in our study indicates that sCD127 is likely not directly mediating the IL-7-mediated effects. Further studies are required to more definitively demonstrate the biological function of this secreted receptor.

The bioavailability limitations of administered IL-7 are typically thought to be mediated by cytokine half-life or its consumption by altered mCD127 expression[27]. In this study, the overall proportion of mCD127^+^ T-cells was similar prior to each IL-7 cluster, hence this was thought not to play a role in mediating IL-7 effects[34], although others have reported that IL-7 administration to macaques results in difficulties in detecting CD127 expressing cells due to CD127 down-regulation and IL-7 blocking of the CD127 receptor[56, 57]. Based on findings by Okoye et al, which demonstrated that naïve, memory, central memory, transitional memory and effector memory CD8^+^ T-cells all express Ki67 in response to IL-7 therapy in both adult and aged macaques[58], one can infer which subsets are responses to the cytokine. Similarly, our data indicated that TN and TEM cell subsets expressed the most Ki67, and hence would presumably be those increasing in association with sCD127 levels (Fig 5B). Dereuddre-Bosquet et al determined that approximately 64% of CD3^+^CD4^+^ and 47% of CD3^+^CD8^+^ T-cells express mCD127 in uninfected, untreated macaques, but that IL-7 therapy resulted in a competition for the detection of CD127^+^ cells[57]. Consideration of the hierarchical expression pattern of mCD127 (i.e. memory cells > naïve cells > effector cells) informs the potential effects of IL-7 on distinct cell subsets[59] and the responsive cells in this study, namely T_N_ and T_CM_ cells, expressed high levels of mCD127 (approx. 70% and 65%, respectively) before SIV infection. The IL-7-induced migration of T-cells from the blood to organs such as lymphoid organs and the gut, as has been reported previously, should also be considered[33]. And lastly, similar to HIV infection, one must contend with the fact that SIV infection also results in the downregulation of mIL-7Rα expression while3 IL-2 receptor γ expression is also decreased[60].

In conclusion, this study has demonstrated that sCD127 concentrations are associated with the immunorestorative outcomes of repetitive IL-7 therapy in SIV-infected Rhesus macaques receiving ART. There was significant evidence that increases in sCD127 correlated with increased T-cell numbers and survival. This suggests that sCD127 may have a role in the replenishment of the T-cell pool with IL-7, although further studies are required. Lastly, the magnitude and frequency of increased concentrations of plasma sCD127 concentrations in > 1 cluster of IL-7 treatment was more pronounced among animals that lost, or lacked viral control. Highlighting such relationships provides insight into the design of immunorestorative strategies for HIV infection, while building an awareness of the effects this may have on “Cure”. Therefore, this study provides insights regarding the ability of sCD127 to be utilized as an indicator of IL-7 associated therapies that might be utilized in future HIV-infected patients.

## Abbreviations:

ART: antiretroviral therapy
CD127: interleukin-7 receptor α
CTL: cytolytic
d.p.i.: days post-infection
FTC: emtricitabine
mCD127: membrane-bound CD127
sCD127: soluble CD127
IL-7: interleukin-7
PBST: phosphate buffered saline-Tween
PMPA: 9-R-2-phosphonomethoxypropyl adenine
rsIL-7gly: glycosylated recombinant simian IL-7
SIV: simian immunodeficiency virus
